# **Who Deserves Welfare and Who Does Not?** Comment on "A Scoping Review of Populist Radical Right Parties’ Influence on Welfare Policy and its Implications for Population Health in Europe"

**DOI:** 10.34172/ijhpm.2020.174

**Published:** 2020-09-13

**Authors:** Jasper Muis

**Affiliations:** Department of Sociology, Vrije Universiteit Amsterdam, Amsterdam, The Netherlands.

**Keywords:** Populist Radical Right, Welfare Policy, Welfare Chauvinism, Public Health

## Abstract

To what extent has the rise of populist radical right (PRR) parties in Europe affected welfare policies? Based on a scoping review of studies that address the relationship between PRR parties and welfare policy, Chiara Rinaldi and Marleen Bekker conclude that, due to their welfare chauvinistic positions, the participation of PRR parties in government coalitions is likely to have negative effects on the access to welfare provisions and health of vulnerable population groups. This short commentary reflects on this review article and critically examines its conclusion. It suggests some conceptual clarifications, raises some reservations about the review’s main claim, and provides some follow-up questions.

The review article of Chiara Rinaldi and Marleen Bekker^1^ explores the welfare policy consequences of the rise of populist radical right (PRR) parties in Europe. It contains 15 articles, of which 11 address the relationship between PRR parties and welfare policy (the other four seem a bit misplaced in this review as they address different research questions). The authors explain that welfare policy revolves around “redistribution of economic prosperity across different groups in society,” (p. 2) but they are particularly interested in population health and health equity; welfare policy acts as proxy for these variables.

Whereas far right ‘core issues,’ most notably migration politics, have received much attention, the impact of PRR incumbency on welfare policies, let alone public health, has barely been explored.^2,3^ The laudable effort of Chiara Rinaldi and Marleen Bekker to summarize what we know thus far is thus very welcome and helpful for the development of future research. Since research on PRR parties’ impact on welfare policy-making is still in its infancy, it is understandable that the review has an explorative character.

The review’s most remarkable conclusion is that “early evidence suggests that the welfare chauvinistic ideology of PRR parties is harmful for public health.” More specifically, the authors conclude that PRR parties’ welfare chauvinistic positions “are likely to have negative effects on the access to welfare provisions and health of vulnerable population groups” (p. 1). This short commentary critically examines and reflects on this conclusion. It suggests some conceptual clarifications, raises some reservations about the review’s main claim, and provides some follow-up questions. One essential element of the review is probably hardly surprising for many readers: it is by now well-established that most Western European PRR parties have adopted a nativist perspective on social policies that is called ‘welfare chauvinism.’^4-6^ This is a combination of support for economic redistribution with resistance towards distributing welfare services for immigrants.^7^ Andersen and Bjørklund have aptly summarized it as the idea that “welfare services should be restricted to ‘our own’” (p. 165).^7^ Hence, PRR parties endorse redistribution, but want this redistribution to be restricted to the native population: they are favouring generosity for natives, whilst advocating cuts and restrictions for immigrants.^4-6^In addition, some (but certainly not all) PRR parties, especially the Swiss SVP and Norwegian FrP, adopted what Simon Otjes and colleagues call ‘economic authoritarianism.’^5^ This implies the viewpoint that the “undeserving poor” should be punished, because their poverty is seen as consequence of their own fault and “moral failing.” It is mainly targeted at “those who can work but prefer to live off social benefits” (p. 274).^5^ This includes both natives and non-natives, but scholars have nevertheless referred to such welfare retrenchment as “indirect welfare chauvinism” when it is motivated by the assumption that immigrants are overrepresented in the particular service (in this case unemployment benefits).^8^

 Several reviewed articles focus on PRR’s ideological positioning and indeed underline that many current PPR parties have increasingly adopted welfare chauvinistic views. Besides comparative studies of Western European countries, the review includes case studies of Austria, Switzerland, Denmark, Sweden, and Finland. Remarkably, it lacks studies on Central and Eastern Europe, which could be an interesting avenue for future studies.

Another important follow-up question discussed in the review is whether PRR parties are successful in translating their preferences into public policy: does the presence of powerful PRR parties lead to alterations of policies? Although they can also influence policy indirectly, by influencing the positions of other parties,^3^ participation in government coalitions is considered the main channel by which PRR parties attempt to feed their viewpoints into the policy-making process.^9^ It therefore makes sense that the review focuses on the consequences of PRR parties’ participation in government coalitions.

 Having said that, I have some reservations about the review’s main conclusion. One reservation stems from the fact that not a single study in the review includes a direct outcome measure of health spending or health policies. Moreover, the review seems to conflate the concepts of ‘population health’ and ‘health equity.’ I would argue that it is pivotal to conceptually distinguish both terms. Translated to the domain of welfare polices: the question how much the government spends on welfare is fundamentally different from how this spending is distributed among different social groups. Generosity in terms of expenditure does not necessarily go hand in hand with generosity in terms of unrestricted, universal access to welfare provisions.

The first issue at stake, which features most prominently in the review, is whether welfare state expenditure comes with limitations concerning who has access to social benefits. This indeed raises a different question than the issue of welfare spending in general: it revolves around equal rights and equal access. The review aptly concludes that access to welfare provisions of certain vulnerable population groups might be negatively affected by the rising political influence of PRR parties. Although the authors do not spell out the vulnerable groups and policy measures in much detail, it becomes evident that primarily non-natives are affected, such as refugees and migrants. For instance, the Danish welfare state has introduced a number of measures that, in principle or in practice, apply differently to Danish citizens and to immigrants.^8,10^We should acknowledge the double-edged nature of PRR parties’ influence on welfare state policies: one would expect that PRR parties’ government participation results in the preservation of the welfare state for population groups that are considered ‘deserving’ of such support and restriction of social benefits for ‘undeserving’ groups. It seems that the elderly and the sick mainly belong to the first category, whereas the unemployed and non-natives belong to the second category.^11^ This idea is confirmed by interesting research by Juliana Chueri – not included in the review.^2,9,12^ Her findings indicate that participation of PRR parties in government coalitions generally lead to the adoption of welfare retrenchment (compared with other types of governments), but social expenditure on unemployment benefits and immigrants’ access to social rights are the main targets of cutbacks. In contrast, public expenditure on pension schemes is not negatively affected by PRR government participation, compared to a mainstream right-wing government. Although the particular coefficient is not statistically significant, the results suggest that PRR government participation “might corroborate to a lesser reduction of old-age expenditure, in comparison with governments of mainstream right-wing parties” (p. 6).^2^

 The main problem of the review’s conclusion is that this empirical confirmation of PRR parties’ welfare chauvinist position does not tell us very much about public health. By definition, this position implies the protection or expansion of welfare for some groups, but restrictions for others. To be able to relate this to “harmful public health effects” would requires a complex weighting of potential negative indirect effects on public health of migrant’s reduced access to welfare (eg, social assistance, healthcare) versus the potential positive effect of increased pensions spending (eg, lower retirement age).

Even if one simply takes the total amount of governmental spending on welfare as proxy for overall population health, as far as I can tell the review does not contain an empirical study that demonstrates an unequivocal negative influence of PRR parties’ incumbency on the overall amount of welfare spending. In fact, interestingly, the quantitative analysis of one of the reviewed articles shows that the presence of PRR parties causes governments to *refrain* from welfare retrenchment.^13^ In that study Leonce Röth and colleagues conclude that right-wing, market-liberal governments with PRR participation are less inclined to pursue welfare retrenchment than right-wing governments without PRR participation.^13^ Hence, based on this particular comparison, PRR parties may in fact have a positive effect on welfare polices.

On the other hand, one can indeed conclude that PRR parties in the government are harmful for welfare policies if they replace a social democratic party as coalition partner, as for instance happened in Austria in 2000. When Haider’s Freedom Party (FPÖ) joined the government with the conservative right (ÖVP), a long tradition of power-sharing between the conservative party and social democratic party ended. Consequently, austerity measures were more easily implemented.^14^ As scholars point out, “building a coalition with the FPÖ was perceived as an opportunity for the ÖVP to push through the retrenchment and deregulation measures which had been watered-down while in government with the social democrats” (p. 343).^13^

To conclude, it is misleading to speak in a general way about PRR parties being “harmful for public health” without specifying the reference condition: harmful *compared to what*? Using the three-fold typology of Esping Anderson, I would expect that the PRR ideology is indeed disadvantageous for social policies compared with social-democratic approaches (eg, Scandinavia), but more generous than liberal approaches (eg, United States).^7^ On the one hand, when PRR parties join right-wing cabinets, they are willing to support welfare cuts and ‘painful reforms,’ probably to have some bargaining power on immigration and integration policies.^9^ On the other hand, PRR parties would ‘betray’ their electoral supporters if they make too many concessions in terms of market-liberal positions.^14^ This ambivalent, in-between position on socio-economic issues of PRR parties^15^ matches the attitudes of their voters: compared with centre-right voters, far-right voters more strongly *support* income redistribution, but compared with supporters of traditional left-wing parties, they more strongly *oppose* income redistribution.^16^

The conditional nature of welfare chauvinism — PRR parties oppose or advocate retrenchment, depending on ‘deservingness’ — naturally leads to discussions about the meaning of social citizenship. The judgement about why some people deserve welfare and others not is rooted in an ethic of membership in a national community. As for instance Will Kymlicka explained, the welfare state is built on national solidarity; it is tied to mutual concerns and obligations we have as members of a shared society.^17^

Regrettably, the review does not touch upon the trade-off between inclusiveness and feelings of solidarity (required for redistribution policies). Some social progressives are concerned that the welfare state and large-scale immigration — and ethnic diversity it brings — are not easily combined. A universal welfare state provides good social protection for immigrants, but is also economically vulnerable to immigration. If certain migrants (such as irregular labour migrants and failed asylum seekers) are able to become full-fledged members of society very easily, solidary systems could become unaffordable and lose support. As Godfried Engbersen succinctly puts it: “To secure solidarity with a country’s own (vulnerable) citizens, others have to be excluded” (p. 2).^18^ Even the ‘universalistic’ Scandinavian welfare states intend to be universalistic within the boundaries of the national political community only.^19^ It should also be noted that ‘strict’ welfare chauvinism — banning immigrants’ access to social services— is rare; more commonly, “people believe immigrants should receive benefits after meeting some preconditions or making some contribution” (p. 187).^12^

Consequently, we seem confronted with a choice between neoliberal multiculturalism — inclusion without solidary — and welfare chauvinism — solidarity without inclusion.^19^ The way out of this so-called ‘progressive’s dilemma’ that is proposed by Will Kymlicka constitutes a third option: a multicultural welfare state (both inclusion and solidarity).^17^ But to what extent this is a realistic possibility? For the sake of completeness, the fourth option that can be mentioned is neoliberal nationalism (exclusion and no solidarity).

The difficulty faced by the vision of a generous and inclusive, multicultural welfare state is that an extensive body of scholarship hypothesizes that immigration undermines public support for social policy.^15^ This hypothesis is not uncontested as research on the issue is divided.^20^ As Stuart Soroka and colleagues note: “In short, the relationship between migration and welfare state spending is complex” and “mediated by a number of factors” (such as population demographics) (p. 176).^20^ They found that it is domain-dependent — particularly, immigration significantly decreases unemployment spending, but most other domains are not affected. Moreover, there are alternative macro-level factors that more importantly shape opinions of the native’s population about distributing welfare to immigrants.^7^ Especially for wealthy and well-educated citizens, immigration has probably a negligible effect on public opinions concerning the welfare state, if any effect at all. Even so, despite these reservations, immigration and growing ethnic diversity affect the opinions of the lower educated, thus the most ‘natural’ supporters of the welfare state.^6^

The progressive’s dilemma has therefore important political implications, as voters who are less inclined to support policies that benefit mostly ethnic minority groups have abandoned left-wing social-democratic parties in favour of radical right-wing parties.^3,6^ The left-progressive ideal of ‘inclusive solidarity’ is apparently not attractive for a large part of the traditional constituency of social-democratic parties, who have turned to PRR parties instead. All in all, for proponents of a generous, multicultural welfare approach that promotes health equity for all, the “million dollar question” remains how to convince the native working class to adjust its narrow, nationalist conceptualisation of social citizenship (p. 3).^19^

Finally, it is important to note that context matters.^7^ What makes the above-mentioned question particularly challenging is that it might require different answers for different national settings, because of country-specific factors or conditional effects. The review discusses several important political system characteristics that potentially influence the degree to which PRR parties succeed to shape welfare policy, such as the political economy and judicial institutions — rather confusingly, Chiara Rinaldi and Marleen Bekker hereby refer to ‘mediation’; the correct term for what they actually describe is ‘moderation’ (interaction). Besides the more stable institutional characteristics discussed in the review, immigration rates and the extent to which immigration-related issues are salient topics in public debate might play a moderating role, too. For instance, the ‘refugee crisis’ in 2015 prompted heated debates in which immigrants were portrayed as cultural threat and economic burden. Such moderating factors could not only explain why some PRR parties more successfully shaped policy reforms than others, but also why mainstream parties in some national contexts more strongly support welfare chauvinism than their counterparts in other contexts.^15^ After all, mainstream right-wing parties have also independently adopted welfare chauvinistic measures, regardless of electoral successes and government participation of PRR parties.^2,13^To conclude, future work could benefit from more fine-grained distinctions addressing the different domains and various types of welfare services (pensions, healthcare, child benefit, unemployment benefits etc) and — rather than try to ‘isolate’ the influence of PRR parties — more systematically explore the interplay of all relevant factors that determine why some citizens and politicians (including the mainstream ones) more strongly support the implementation of welfare chauvinist policies than others.

## Acknowledments

JM would like to thank the two anonymous reviewers of *IJHPM* very much for their valuable feedback.

## Ethical issues

 Not applicable.

## Competing interests

 Author declares that he has no competing interests.

## Author’s contribution

 JM is the single author of the paper.

## References

[1] Rinaldi C, Bekker MPM (2020). A scoping review of populist radical right parties’ influence on welfare policy and its implications for population health in Europe. Int J Health Policy Manag.

[2] Chueri J (2020). Social policy outcomes of government participation by radical right parties. Party Politics.

[3] Krause W, Giebler H (2020). Shifting welfare policy positions: the impact of radical right populist party success beyond migration politics. Representation.

[4] Fenger M (2018). The social policy agendas of populist radical right parties in comparative perspective. J Int Comp Soc Policy.

[5] Otjes S, Ivaldi G, Jupskås AR, Mazzoleni O (2018). It’s not economic interventionism, stupid! reassessing the political economy of radical right-wing populist parties. Swiss Polit Sci Rev.

[6] van der Waal J, Achterberg P, Houtman D, de Koster W, Manevska K (2010). ‘Some are more equal than others’: economic egalitarianism and welfare chauvinism in the Netherlands. J Eur Soc Policy.

[7] van Der Waal J, de Koster W, van Oorschot W (2013). Three worlds of welfare chauvinism? how welfare regimes affect support for distributing welfare to immigrants in Europe. Journal of Comparative Policy Analysis: Research and Practice.

[8] Careja R, Elmelund-Præstekær C, Baggesen Klitgaard M, Larsen EG (2016). Direct and indirect welfare chauvinism as party strategies: an analysis of the Danish people’s party. Scan Polit Stud.

[9] Chueri J. Who Deserves the Welfare? The Populist Radical Right’s Transformation of Social Policy [thesis]. Geneva: University of Geneva; 2020.

[10] Andersen JG (2007). Restricting access to social protection for immigrants in the Danish welfare state. Benefits.

[11] van Oorschot W (2006). Making the difference in social Europe: deservingness perceptions among citizens of European welfare states. J Eur Soc Policy.

[12] Chueri J. Who’s to blame: radical right populist party and mainstream parties’ roles in adoption of welfare chauvinist policies. In: Biard B, Bernhard L, Betz HG, eds. Do They Make a Difference? The Policy Influence of Radical Right Populist Parties in Western Europe. London: ECPR Press; 2019:185-222.

[13] Röth L, Afonso A, Spies DC (2018). The impact of populist radical right parties on socio-economic policies. Eur Polit Sci Rev.

[14] Afonso A (2015). Choosing whom to betray: populist right-wing parties, welfare state reforms and the trade-off between office and votes. Eur Polit Sci Rev.

[15] Spies DC. Immigration and Welfare State Retrenchment: Why the US Experience is Not Reflected in Western Europe. Oxford: Oxford University Press; 2018.

[16] Brils T, Muis J, Gaidytė T (2020). Dissecting electoral support for the far right: a comparison between mature and post-communist European democracies. Gov Oppos.

[17] Kymlicka W (2015). Solidarity in diverse societies: beyond neoliberal multiculturalism and welfare chauvinism. Comp Migr Stud.

[18] Engbersen G (2016). Floating populations, civic stratification and solidarity: comment on Will Kymlicka’s article: “Solidarity in Diverse Societies”. Comp Migr Stud.

[19] Kriesi H (2015). Enlightened understanding, empowerment and leadership - three ways to enhance multiculturalism: comment on Will Kymlicka’s article: “Solidarity in Diverse Societies”. Comp Migr Stud.

[20] Soroka SN, Johnston R, Kevins A, Banting K, Kymlicka W (2016). Migration and welfare state spending. Eur Polit Sci Rev.

